# Symptoms of respiratory tract infection and associated care-seeking in subjects with and without obstructive lung disease; The Tromsø Study: Tromsø 6

**DOI:** 10.1186/1471-2466-12-51

**Published:** 2012-09-07

**Authors:** Hasse Melbye, Lisa Joensen, Mette Bech Risør, Peder A Halvorsen

**Affiliations:** 1 General Practice Research Unit, Department of Community Medicine, Faculty of Health Science, University of Tromsø, MH-building, 9037, Tromsø, Norway

**Keywords:** Respiratory tract infection, Care-seeking, Consultation rates, Obstructive pulmonary disease, Antibiotics, Self-medication

## Abstract

**Background:**

Respiratory tract infections (RTIs) may be more severe in those with asthma or COPD and these patients are more frequently in need of health care. The aim of the study was to describe the frequency of RTI symptoms in a general adult population and how care-seeking is associated with the presence of obstructive lung disease.

**Methods:**

Cross-sectional data including spirometry and self-reported chronic diseases were collected among middle-aged and elderly subjects in the Tromsø population survey (Tromsø 6). Self- reported RTI symptoms, consultations and antibiotic use were the main outcome variables. Possible predictors of RTI symptoms were evaluated by multivariable logistic regression.

**Results:**

Of the 6414 subjects included, 798 (12.4%) reported RTI symptoms in the previous week. RTI symptoms were reported less frequently by subjects aged 75 years or above, than by those younger than 55 years (OR 0.5). Winter season (OR 1.28), current smoking (OR 1.60), low self-rated health (OR 1.26) and moderate to severe bronchial obstruction (OR 1.51), were also statistically significant independent predictors of RTI symptoms, but these variables did not predict RTI symptoms that had started within the previous seven days. Among subjects with RTI symptoms, 5.1% also reported a consultation with a doctor. In those with bronchial obstruction by spirometry, who did not report asthma or COPD, this frequency was 2.4%. Antibiotic treatment was reported by 7.4% of the participants, among whom one third had consulted a doctor. Antibiotics were taken more frequently when asthma or COPD was reported (13.7%), but not in subjects with bronchial obstruction who did not report these diseases (7.2%).

**Conclusions:**

RTI symptoms seldom led to consultation with a doctor and not even in subjects with obstructive lung disease. This was in particular the case in subjects who did not know about their obstructive lung disease. Strategies for early diagnosis of COPD and providing health care to subjects with such disease cannot rely on their doctor visits due to respiratory symptoms.

## Background

Symptoms of possible respiratory tract infections (RTIs), such as cough, sore throat and rhinitis are common in the community. In the USA, 19% of an adult population reported to have had a cough, cold or another acute illness in the previous few days [1]. In Norway 13.8% of an elderly population reported symptoms of airway infection within the last 3 weeks [2]. Symptoms of airway infection are frequent reasons for seeking health care [3], but most subjects with a possible RTI do not visit their family doctor. A consultation rate just below 10% was found among women in UK aged 16–44 years with a cold, a flu or sore throat [4], whereas 25.4% of subjects reporting RTI in the Tecumseh study (USA) had consulted a doctor [5]. Among the 22% of an adult population who reported symptoms of RTI the last 2 weeks in a Dutch survey, 25% visited a general practitioner (GP) [6]. The consultation rate is higher for influenza than for common cold [5]. During the swine flu epidemic in the USA 2009–10, 8.1% of adults reported the “flu” in the last 30 days; among these 40% sought health care [7].

In Sweden, consultation rates for upper RTIs (URTIs) has shown a decline since 1999, but have remained unchanged for influenza and lower RTI [8]. Similar findings have been found in UK, based on patient records from general practices [3]. The decline in consultation rate has been explained by a more restrictive prescribing of antibiotics teaching patients that visits to the doctor are often unnecessary. The rate of prescribing antibiotics per RTI consultation has been rather stable in Sweden [8]. Notably in the UK, a decline in URTI related antibiotic prescribing was observed between 1997 and 2006 [8].

While RTIs for most of us are uncomplicated events, some may have a more severe clinical course. This is particularly the case for patients with obstructive pulmonary diseases [9]. Frequent exacerbations are associated with a more rapid decrease in lung function, reduced quality of life, and severe exacerbations often lead to acute hospital admissions. This represents a great burden, both for the individual and the society [10]. Such patients should probably consult a GP more frequently during RTIs than those who are otherwise healthy, as early treatment is important for a favourable outcome [11].

The aim of this study was to describe the frequency of RTI symptoms in a general population, the proportion of people with RTI symptoms that consult a doctor, and how often such illnesses are treated with antibiotics. We also wanted to find out to what degree the consultation rate and use of antibiotics are associated with self-reported obstructive pulmonary disease or reduced pulmonary function, as measured by spirometry.

## Methods

### Subjects

The subjects studied were participants in the Tromsø Study, a repeated epidemiological, prospective study of health problems, symptoms and chronic diseases in the city of Tromsø, dating back to 1974 [12]. Tromsø is a city in the northern part of Norway with 69,000 inhabitants. The sixth survey was conducted between October 2007 and December 2008, with a break in July 2008. The Department of Community Medicine, University of Tromsø, oversees the scientific leadership and administration of the Tromsø Study.

Subjects invited to participate in Tromsø 6, included all residents in some age groups and random samples of others (Table 1) The Population Registry of Norway, with a unique national identity number given to all citizens, was the source for the invitations. The attendance rate was 65.7%, for the first visit, and about two-thirds of those who attended the first visit were invited to a second visit with more extended medical examination (Table 1), in which most of the important examinations relating to this study took place. 7307 (91.8%) attended this second visit.

**Table 1:** Subjects invited to participate in the Tromsø 6 study

**First visit**	
Age 30 – 39	10% random sample
Age 40 – 42	All residents
Age 43 – 59	40% random sample
Age 60 – 87	All residents
Attenders to the second visit of Tromsø 4^#^	All residents if not included above
**Second visit**	
Age 50 – 62	All attenders to first visit
Age 63 – 74	20% random sample of attenders to first visit
Age 75 – 84	All attenders to first visit
Attenders to the second visit of Tromsø 4^#^	All attenders to first visit (in Tromsø 6)

^#^ The whole Tromsø population aged 25 years or more were invited to Tromsø 4 in 1994, and all the attending men aged 55–74 years and the attending women aged 50–74 years were invited to the second visit [12].

### Examinations

Before attending the first visit, participants had to fill in a questionnaire on health issues including self-rated health, previous diseases and smoking habit. Participants reporting angina pectoris, myocardial infarction, cerebral stroke or atrial fibrillation were classified as “self-reported cardiovascular disease”. Height and weight were measured during the first visit. Other characteristics of the participants are dealt with in a previous publication [13].

At the spirometry station during the second visit, a questionnaire concerning recent symptoms of possible RTI and subsequent consultations with doctors and antibiotic treatment was filled in:

• Have you had symptoms of the common cold, acute bronchitis or any other symptoms of airway infection in the previous 7 days?

• If you have had symptoms of the common cold, acute bronchitis or any other airway infection in the previous 7 days, how many days have passed since this illness started?

• If you have had symptoms of the common cold, acute bronchitis or any other airway infection the previous 7 days, have you consulted a doctor (your GP, a GP on-call, a respiratory physician , or “other doctor”) for this illness?

• If you have had symptoms of the common cold, acute bronchitis or any other airway infection in the previous 7 days, have you taken antibiotics for this illness?

They were also asked whether they had taken any drugs for asthma or COPD that same day. This questionnaire was computerised and each question had to be answered before moving to the next.

Spirometry was performed by trained technical staff with the use of a “Sensor Medics Vmax Encore 20” spirometer, following ATS/ERS criteria [14]. Post-bronchodilator spirometry was not carried out. Norwegian reference values were used [15]. The spirometry results were only found acceptable for analysis in subjects who expired for more than 3 seconds and with a forced expiratory volume in one second (FEV1) >0.3 l. To avoid misclassifying subject with normal lung function as obstructive, those with FEV1/FVC <0.7 or FEV1 < 80% predicted were excluded from analysis if expiration was not carried out with sufficient force, defined by a peak expiratory flow (PEF) ≥ 3 x forced expiratory flow when 75% of the air had been expired (FEF75).The spirometry results were categorized according to the Global Initiative for Chronic Obstructive Lung Disease (GOLD) classification [9]. Due to few subjects with severe obstruction, participants with moderate and severe obstruction (FEV1 <80% predicted combined with a FEV1/forced vital capacity (FVC) ratio <0.7) were merged into one group for the main analysis, named here as “GOLD 2–4 spirometry”. Mild COPD was defined as FEV1/FVC <0.7 and FEV1 ≥ 80% predicted (GOLD 1 spirometry).

### Statistical analyses

The proportion reporting recent RTI symptoms, consultations with doctors, antibiotic treatment as well as the duration of the symptoms, were analysed by characteristics of the participants, such as smoking habit, self-reported diseases and spirometry results. The groups of subjects reporting asthma or COPD (chronic bronchitis/emphysema/COPD), were merged into “self-reported asthma or COPD” in some analyses, due to the high frequency of COPD in patients reporting asthma, and in order to focus on differences between subjects with and without an established diagnosis of obstructive lung disease. To control for the variation in illness duration between different subgroups, and as an indication of the weekly incidence of new RTI symptoms, RTI symptoms that had appeared within the previous 7 days was also applied as outcome variable. Differences between groups were analysed by Chi-square, Mann–Whitney, and Jonckheere-Terpstra tests. Gender, age, and the possible predictors of RTI in bivariate analysis were entered a multivariable logistic regression, with self-reported RTI symptoms as outcome variable. Similarly, variables significantly associated with days since the symptoms started, were evaluated as predictors of illness duration by multivariable linear regression. SPSS 19.0 for Windows (IBM SPSS Statistics, USA) was used in the statistical analyses. The Tromsø Study complies with the Declaration of Helsinki and each subject gave written informed consent prior to participation. The Regional Committee of Medical and Health Research Ethics approved the study.

## Results

The questionnaire concerning symptoms of possible RTI was completed by 6414 participants, 80.6% of those invited. Spirometry was found acceptable in 6305 subjects, (92.8% of these expired for 6 seconds or more). There were also missing data concerning smoking (n = 91) and self-rated health (n = 61). The mean age was 63.7 years of age, 57.0% were women. COPD and asthma were reported by 5.2% and 10.0% respectively. FEV1/FVC <0.7 was found in 1422 subjects (22.5%), in 59.6% of those who reported COPD, in 37.3% where asthma, but not COPD, was reported and in 19% of the remaining subjects. A total of 413 subjects reported medication for asthma or COPD taken on the examination day. Among these, 89% also reported asthma or COPD, 4% belonged to the subgroup with FEV1/FVC <0.7 who did not report asthma or COPD, whereas 7% belonged to none of these subgroups.

Of the 6414 individuals, 798 (12.4%, 95% CI 11.8%-13.0%) reported symptoms of RTI from within the previous seven days, with the highest rate in December (16.9%) and the lowest rate in June (9.1%). In addition, increased reporting of recent RTI symptoms was found in current smokers and in those reporting reduced self-rated health. Spirometry signs of bronchial obstruction was strongly associated with recent RTI symptoms, also when asthma or COPD were not reported (Table 2). Age of 75 years or above was associated with less frequent reporting of RTI symptoms. In multivariable logistic regression, the same five variables mentioned above remained statistically significant predictors of recent RTI symptoms (Table 3).

**Table 2:** Frequency of reporting symptoms of respiratory tract infection last week by characteristics of 6414 middle-aged and elderly adults taking part in the Tromsø 6 survey

	**Symptoms of RTI last week**			**Days since start of symptoms**		**Symptoms of RTI last week that started within the previous 7 days**		
	**n**	**%**	**p-value**	**median**	**p-value**	**n**	**%**	**p-value**
**All** (n = 6414)	798	12		8		340	5.3	
**Gende**r								
Male (n = 2754)	350	13	0.6	8	0.6	149	5.4	0.7
Female (n = 3660)	448	12		8		191	5.2	
**Age**								
<55 years (n = 1059)	141	13	0.001^#^	7	0.03^£^	79	7.5	<0.001^#^
55-64 years (n = 2656)	354	13		9		138	5.2	
65-74 years (n = 1752)	230	13		9		217	5.5	
≥ 75 Years (n = 947)	947	8		14		123	2.7	
**Smoking** (n = 6323)								
Current (n = 1158)	214	19	<0.001^#^	11	0.01^£^	76	6.6	0.7
Previous (n = 2955)	334	11		8		132	4.5	
Never (n = 2210)	240	11		7		126	5.7	
**Season**								
Winter (November-April) (n = 3128)	425	14	0.007	9	0.2	175	5.6	0.3
Summer (May-October) (n = 3286)	373	11		8		165	5.0	
**Self-reported disease**^**&**^								
Asthma, not COPD (n = 457)	69	15	0.07	11	0.5	28	6.1	0.4
COPD, not asthma (n = 172)	25	15	0.4	14	0.09	6	3.5	0.3
Both asthma and COPD (n = 141)	22	16	0.3	12	0.5	9	6.4	0.6
Neither asthma nor COPD (n = 5578)	679	12	0.09	8	0.1	296	5.3	1.0
Cardiovascular disease (n = 1154)	134	12	0.3	12	0.02	47	4.1	0.04
**Self-rated health** (n = 6353)								
Good/exellent (n = 3949)	449	11	0.001^#^	8	0.01^£^	207	5.2	0.9
Neither good nor bad (n = 2057)	288	14		10		109	5.3	
Bad/very bad (n = 347)	53	15		14		19	5.5	
**Lung function** (n = 6305)								
Normal/restrictive (n = 4881)	567	12	<0.001^#^	8	<0.001^£^	267	5.5	0.2^#^
GOLD 1 (n = 643)	80	12		11		26	4.0	
GOLD 2–4 (n = 781)	134	17		13		31	4.6	
**Self-reported asthma or COPD and lung function combined** (n = 6305) ^**&**^								
Asthma or COPD not reported, FEV1/FVC ≥ 0.7 (n = 4436)	511	12	0.001	8	0.009	235	5.3	0.7
Asthma or COPD reported, FEV1/FVC ≥ 0.7 (n = 445)	56	13	0.9	7	0.09	32	7.2	0.05
Asthma or COPD reported, FEV1/FVC <0.7 (n = 380)	61	16	0.03	14	<0.001	11	2.9	0.04
Asthma or COPD not reported but GOLD 1 spirometry (n = 559)	69	12	1.0	9	0.9	25	4.5	0.4
but GOLD 2–4 spirometry (n = 485)	84	17	0.001	12	0.04	26	5.4	0.9

^#^Chi-square linear trend.

^**&**^Subject with and without the condition are compared.

^£^ Jonckheere-Terpstra test.

**Table 3:** Predictors of self-reported symptoms of airway infection the previous seven days (n = 763), and that had started within the previous seven days (n = 319), evaluated by multivariable logistic regression in 5943 middle-aged and elderly adults taking part in the Tromsø 6 survey

	**RTI symptoms previous week**			**RTI symptoms that started within previous week**		
	**Odds ratio**	**95% confidence interval**	**p-value**	**Odds ratio**	**95% confidence interval**	**p-value**
**Male gender**	1.06	0.90 - 1.24	0.5	1.13	0.90 - 1.43	0.3
**Age** (years)^#^ : 55-64	0.95	0.76 - 1.17	0.6	0.67	0.50 - 0.90	0.008
65-74	0.94	0.74 - 1.19	0.6	0.77	0.56 - 1.06	0.1
75 +	0.49	0.35 - 0.68	<0.001	0.35	0.21 - 0.57	<0.001
**Smoking**^**&**^ : current	1.60	1.29 - 1.98	<0.001	1.12	0.82 - 1.54	0.5
previous	0.99	0.82 - 1.19	0.9	0.80	0.61 - 1.03	0.09
**Self-rated health**, bad , or neither good or bad	1.30	1.11 - 1.53	0.001	1.12	0.88 - 1.42	0.4
**Self-reported disease:** Asthma, not COPD^£^	1.22	0.93 - 1.62	0.2	1.22	0.81 - 1.85	0.3
COPD, not asthma^£^	0.97	0.60 - 1.55	0.9	0.72	0.29 - 1.81	0.5
Both asthma and COPD^£^	1.06	0.65 - 1.64	0.8	1.33	0.63 - 2.81	0.5
Cardiovascular disease	0.91	0.73 - 1.13	0.4	0.79	0.56 - 1.12	0.2
**Spirometry**^**$**^ : GOLD 1 spirometry	1.08	0.83 - 1.40	0.6	0.72	0.46 - 1.13	0.2
GOLD 2–4 spirometry	1.48	1.17 - 1.87	0.001	0.89	0.60 - 1.33	0.6
**Winter season**	1.27	1.09 - 1.50	0.002	1.15	0.92 - 1.45	0.2

The area under curve for the two models were 0.60 (0.58-0.62) and 0.60 (0.57-0.63), respectively.

^#^ Age 38–54 years is reference category.

^**&**^ Never smoking is reference category.

^£^ Asthma or COPD not reported is reference category.

^**$**^ FEV1/FVC ≥ 0.7 is reference category.

The median number of days since the symptoms began was 8 days. Increased number of days was associated with age, smoking, cardiovascular disease, reduced self-rated health and reduced lung function (Table 2). When analysed by linear regression, cardiovascular disease was the only significant predictor of the duration of illness (p = 0.04, Table 4). RTI symptoms had started within the previous seven days in 340 patients. Such new RTI symptoms was associated with age and absence of cardiovascular disease (Table 2). In logistic regression, age above 55 years was associated with decreased reporting of such new symptoms (Table 3).

**Table 4:** **Predictors of duration of illness as evaluated by linear regression in 757 subjects reporting symptoms of respiratory tract infection the previous week in the Tromsø 6 survey (r**^**2**^ **= 0.03)**

	**Standardized Beta**	**p-value**
**Male gender**	0.05	0.2
**Age**	0.03	0.4
**Smoking**: Current	0.05	0.3
previous	−0.01	0.8
**Self-rated health**: bad or neither good or bad	0.05	0.2
**Self-reported disease**: Asthma, not COPD	0.06	0.1
COPD, not asthma	0.2	0.6
Asthma or COPD	−0.01	0.8
Cardiovascular disease	0.08	0.04
**Spirometry**: GOLD 1 spirometry	−0.01	0.8
GOLD 2–4 spirometry	0.01	0.7

A consultation with a doctor for their RTI symptoms was reported by 41 subjects (5.1%, 95% CI 4.7% - 5.5%)), 32 of these had consulted their GP. Poor self-rated health and a period of more than 14 days since the onset of symptoms, were significantly associated with an increased consultation rate (Table 5). A decreased consultation rate of 2.4% was found among those with FEV1/FVC ratio < 0.7 who did not report asthma or COPD. Taking antibiotics was reported by 58 subjects (7.4%, 95% CI 6.9% - 7.9%). This was found to be more frequent among those with self-reported asthma or COPD (13.7%) and when the symptoms had started more than two weeks earlier, 16.6%, (Table 3). Of those who had taken antibiotics, only one third (17 subjects) reported to have consulted a doctor. When a doctor was consulted, 41% were treated with antibiotics. Among the subjects who did not consult a doctor, antibiotics were taken significantly more frequently where asthma or COPD was reported, in 10.2% (11 subjects), compared to 4.7% amongst the other participants with RTI symptoms, p = 0.02.

**Table 5:** Frequency of consulting a doctor and taking antibiotics in 798 subjects reporting symptoms of respiratory tract infection the previous week in the Tromsø 6 survey

		**Consulting a GP**		**Taking antibiotics**	
	**n**	**n**	**%**	**n**	**%**
**All**	798	41	5.1	58	7.4
**Gender**					
Male	350	19	5.4	22	6.3
Female	448	22	4.9	36	8.0
**Age**					
<45 years	141	4	2.8	8	5.9
55-64 years	354	20	5.6	24	6.8
65-74 years	230	14	6.1	20	8.7
≥ 75 Years	73	3	4.1	6	8.2
**Smoking**					
Current	214	12	5.6	12	5.6
Previous	334	19	5.7	27	8.1
Never	240	10	4.2	18	7.5
**Season**					
Winter	423	17	4.0	23	5.4^£^
Summer	363	24	6.4	35	9.4
**Self-rated health**					
Good/exellent	449	17	3.8^&^	29	6.6
Neither good nor bad	288	19	6.6	22	7.7
Bad/very bad	53	5	9.4	7	13.5
**Self-reported disease**					
Asthma or COPD Yes	119	9	7.6	16	13.7 ^#^
No	679	32	4.7	42	6.3
Cardiovascular disease Yes	134	11	8.2	14	10.5
No	664	30	4.5	44	6.7
Hypertension Yes	269	14	5.2	17	6.4
No	529	27	5.1	41	7.9
**Days since the symptoms started**					
≤ 2 weeks	593	13	2.2	25	4.3
>2 weeks	205	28	13.7^μ^	33	16.6^μ^
**Lung function (n = 794)**					
GOLD 1 spirometry	79	5	6.3	6	7.6
GOLD 2–4 spirometry	134	5	3.7	12	9.1
FEV1/FVC ≥ 0.7	567	31	5.5	40	7.1
**Self-reported asthma or COPD and lung function combined**					
No asthma or COPD reported, FEV1/FVC ≥ 0.7	511	27	5.3	32	6.3
Asthma or COPD reported, FEV1/FVC ≥ 0.7	56	4	7.1	8	14.3
Asthma or COPD reported, FEV1/FVC < 0.7	61	5	8.2	8	13.1
No asthma or COPD reported, but GOLD 1 spirometry	68	3	4.4	4	5.9
No Asthma or COPD reported, but GOLD 2–4 spirometry	84	2	2.4	6	7.1

^£^The difference between the two groups is statistically significant, p = 0.03.

^&^ The variation between the three groups is statistically significant (chi-square linear trend), p = 0.03.

^#^ The difference between the two groups is statistically significant, p = 0.005.

^μ^ The difference between the two groups is statistically significant, p < 0.001.

Among the participants with FEV1/FVC <0.7, those reporting asthma or COPD were more frequently ex-smokers and reporting poor health, compared to those who did not report asthma or COPD (Table 6).

**Table 6:** Characteristics of subgroups of the study population (n = 6305) in %, based on spirometry and self-reported asthma or COPD

	**Male gender**	**Age 65 years or more**	**Former smokers**	**Current smokers**	**Self-reported cardiovascular disease**	**Bad/very bad self-rated health**
	**%**	**%**	**%**	**%**	**%**	**%**
No asthma or COPD reported, FEV1/FVC ≥ 0.7 (n = 4436)	42.1	36.8	46.1	14.4	15.6	3.9
Asthma or COPD reported, but FEV1/FVC ≥ 0.7 (n = 445)	30.8	35.5	46.9	17.5	18.9	14.4
Asthma or COPD reported, and FEV1/FVC <0.7 (n = 380)	44.4^#^	61.9	57.2^&^	26.9^£^	28.1	13.9^&^
Asthma or COPD not reported but GOLD 1 (n = 559)	47.8	60.5	47.1	27.3	20.1	4.9
but GOLD 2–4 (n = 485)	55.3	57.5	45.6	38.1	27.6	5.4

^#^ p = 0.02, ^&^ p < 0.001, ^£^ p = 0.05: statistical significance of the differenece between those who did and who did not report asthma or COPD amongst the participants with FEV1/FVC ratio <0.7 (GOLD 1–4 spirometry).

## Discussion

Symptoms of RTI in the previous week were reported by 12.4%. Somewhat higher frequencies have been found in previous studies [1,2,6]. The relatively low frequency of reported RTI symptoms may be explained by a low occurrence of influenza in Norway during the study period (2007–2008) [16]. The mild influenza outbreak may also explain the moderate fluctuation in the frequency of RTI symptoms throughout the year. We found reduced frequency in the elderly, as did van Duijn and coworkers [6]. The participants who were 75 years of age or older attributed most strongly to the reduced frequency in our study, and a poorer recall among the eldest patients, may possibly explain some of the age effect. Current smoking and GOLD 2–4 spirometry were also strongly associated with the reporting of RTI symptoms. The main reason for this was possibly the longer duration of symptoms in subjects who smoked or had obstructive disease and not an increased incidence of new symptoms, since RTI symptoms that had started in the previous week were not over-represented in these subgroups. Increased reporting of RTI symptoms has previously been found in patients with severe COPD [17], but such patients represented too small a percentage of the subjects with self-reported asthma/COPD or undiagnosed bronchial obstruction, to have had an impact on the results.

Among the 1424 subjects with FEV1/FVC <0.7, only 380 (26.7%) reported to have asthma or COPD. This cannot be explained by incorrect reporting of diseases, since only 4% of those who reported taking respiratory medicine the examination day belonged to the undiagnosed subgroup with bronchial obstruction. Similar frequencies of undiagnosed bronchial obstruction has been found in previous population based studies [18-20]. This study adds that subjects with undiagnosed bronchial obstruction with pre-bronchodilator FEV1/FVC <0.7 and FEV1 <80% predicted, experience prolonged RTI symptoms, similar to subjects with an established diagnosis of asthma or COPD.

Only 5.1% with RTI symptoms had visited a doctor. This is a considerably lower frequency than reported in previous studies [4-6] and may mirror the trend of decreased consultation rates for RTI found in other countries, such as Sweden and the UK [3,8]. The low frequency of influenza in the study period [16] may also have contributed to the low consultation rate. The influenza virus tends to give more severe symptoms than other respiratory viruses [21], with a high consultation rate as a result [5,7]. We found particularly low care-seeking in the winter season. May be people find it more acceptable to have RTI in winter than during other seasons?

The consultation rate among those with undiagnosed bronchial obstruction was remarkably low, just 2.4%. The members of this subgroup had, to a lesser degree, given up smoking and seldom reported bad health, compared to those with bronchial obstruction who reported asthma or COPD. They may have had an increased tendency to postpone health care. Health contacts are not merely a result of individual symptom recognition. Bodily sensations become symptoms only when interpreted as such by the patient Potentially alarming symptoms are sometimes contained in specific social situations [22] and related to cultural values and explanations [23], regarding what is illness and what is poor health [24]. A low consultation rate combined with good self-rated health, despite (although undiagnosed) bronchial obstruction and increased duration of RTI symptoms, points to contextual determinants of symptom interpretation and care-seeking. Reluctance to be confronted with advice to stop smoking may, for instance, have led to a decreased preference of visiting a doctor [25].

Detecting COPD among patients with RTI in primary care, has been suggested as a strategy for case-finding [26]. The low frequency of consulting a doctor among those with undiagnosed bronchial obstruction in the present study, supports that a more comprehensive approach should be applied for early diagnosis of COPD [27].

The prescribing rate when a doctor was consulted (41%) was as expected in Nordic countries [8,28]. The majority of those who had taken antibiotics, did not report to have visited a doctor. A quarter of these belonged to the subgroup with self-reported asthma or COPD, who probably had antibiotics for self-administration at home. Some might have contacted a doctor by phone and received a prescription that way, whilst others may have taken residual tablets from previous prescriptions. Self-medication with antibiotics occurs to a varying extent throughout Europe [29]. In the northern and western parts it is almost impossible to buy antibiotics directly from the pharmacy, and self-medication from left-overs is the most likely way self-medication occurs [29,30]. It is likely that this had happened in our study population as well.

Reducing the prescription of antibiotics is a major target in combating the emerging resistance among human pathogens [28]. Still, it is important that those who actually need antibiotics get their medication without delay. RTIs are the main cause of exacerbations among patients with COPD [9] and often requires treatment with antibiotics [11]. It is well known that patients with COPD often are given prescriptions for short courses of oral steroids and antibiotics for self-administration during acute exacerbations, but the benefit of such practice is questionable [31].

### Strengths and limitations

Our study had the strength of being a population based survey with a high attendance rate, with information obtained directly from the subjects about their symptoms and what they actually did when symptoms occurred. Some age groups were more strongly represented in the study sample than others. The analyses by age groups indicates that this selection probably had little impact on the main results, and adjustment for age has been done in the logistic regression. It has been shown that participants in the Tromsø study are somewhat healthier than non-participants, and a too low frequency of RTI symptoms may have been found.

Post-bronchodilator spirometry was not carried out, and all subjects with FEV1/FVC <0.7, or even GOLD 2–4 spirometry, do not really have COPD or a combination of asthma and COPD.

Some conditions associated with our study, may have contributed to the low care-seeking rate reported. The participants were invited to an extended medical examination, and some with RTI symptoms might have postponed a visit to their own doctor, relying on their forthcoming medical check-up. This statement is supported by the fact that many of the participants expressed confidence that “everything” had been checked when they attended. We do not have information about symptoms among the non-participants. It is likely that some of these felt too ill to attend and might have contacted their own doctor to a greater extent, than those who felt well enough to participate.

## Conclusions

The low frequency of care-seeking due to RTI symptoms may reflect trends of decreasing consultation rates, which has been registered in other countries. To reduce unnecessary use of antibiotics, consulting rates for uncomplicated respiratory infections should be kept low, but without scaring off those who will really benefit from medical care. The consultation rate among subjects with obstructive lung diseases seems to be too low, at least among those who have not got their disease diagnosed. Strategies for early diagnosis of COPD and providing health care to subjects with such disease, cannot rely on their doctor visits due to respiratory symptoms and must therefore be directed towards the public.

## Competing interest

None to declare.

## Authors’ contribution

HM designed the study, led the collection of data, performed the statistical analysis and wrote the final manuscript, LJ contributed to the collection of data, the statistical analysis, and to the drafting of the manuscript. MBR and PAH contributed to the analysis and the final manuscript. All authors have read and approved the final manuscript.

## Pre-publication history

The pre-publication history for this paper can be accessed here:

http://www.biomedcentral.com/1471-2466/12/51/prepub
